# Divergence of the Response Induced by Xenogenic Immunization in the Sepsis Survival of Rats

**DOI:** 10.1371/journal.pone.0125472

**Published:** 2015-05-18

**Authors:** Magdiel Perez-Cruz, Cristina Costa, Rafael Manez

**Affiliations:** 1 Infectious Diseases and Transplantation Division, Bellvitge Biomedical Research Institute (IDIBELL), L’Hospitalet de Llobregat, Barcelona, Spain; 2 Intensive Care Department, Bellvitge University Hospital, L’Hospitalet de Llobregat, Barcelona, Spain; School of Medicine, University of Belgrade, SERBIA

## Abstract

We have previously described that boosted natural xenoantibodies in rats cross-react to bacteria by targeting carbohydrate antigens. This type of immunization is associated with reduced survival after cecal ligation and puncture (CLP). In the present study, we investigated further this phenomenon by immunizing Lewis rats with three intraperitoneal injections, every other day, of hamster blood compared to saline-injected control animals. One day after the last injection, CLP was performed to produce a low-grade sepsis. Induction of xenoantibodies was associated with a reduction in animal survival after CLP relative to controls (45% vs. 90%, p<0.01). No bacterial blood load was observed after CLP in this model either with or without xenoantibody enhancement, indicating that the augmented mortality was not mediated by a direct effect of boosted xenoantibodies over blood bacteria. Nevertheless, the xenoimmunization produced a systemic inflammatory response in all rats. Additionally, a lack of weight gain at the time of CLP was present in animals that died after the procedure, which was not observed in surviving rats and controls. The cytokine profile at the time of CLP in animals that died after the procedure was characterized by an increase in the serum level of several cytokines, particularly adipokines. In contrast, the cytokine profile at CLP of xenoimmunized rats that survived the procedure was characterized by a reduction in the level of cytokines. In conclusion, this study failed to show a direct effect of boosted xenoantibodies over blood bacterial isolates as cause for the decreased survival after CLP. However, it evidenced that non-infectious systemic inflammation may lead to a pattern of augmented cytokines, particularly adipokines, which impairs survival after subsequent CLP. Therefore, the profile of cytokines existing before the infectious insult appears more crucial than that resulting from the condition for the outcome of sepsis.

## Introduction

Natural antibodies are characterized by their recognition of antigens in the absence of any evidence of exogenous exposure to them. These antibodies are mainly IgM, are polyreactive, exhibit modest antigen binding affinity, are directed against carbohydrate antigens, and are due to the direct stimulation of antibody production by T-cell-independent (TI) pathways involving B-1 lymphocytes [1]. Natural antibodies recognize self, altered-self and foreign antigens, comprising an important first line of defense against invading pathogens. Thus, natural IgM antibodies play a critical role in bacterial clearance [2], making mice lacking this isotype of antibodies more susceptible to cecal ligation and puncture (CLP) [3]. In addition, natural antibodies are also important for tissue homeostasis and for inhibiting or preventing inflammatory reactions [4]. Natural IgM antibodies contribute to the removal of apoptotic and transformed cells through a complement-dependent pathway, suppression of inflammation, elimination of altered proteins, and control of autoreactive IgG and antibody-producing B cells capable of causing diseases [5].

Natural antibodies comprise a subgroup that bind antigens depicted on cells and tissues of dissimilar species defined as xenoantibodies [6]. These antibodies appear to be produced in response to bacteria contained in the gut that cross-react with structurally alike xenoantigens. In humans, xenoantibodies include IgM and IgG directed against the galactose α1,3 galactose (αGal) carbohydrate epitope [7], which is expressed in most mammalian species. Anti- αGal antibodies react with various bacteria, including strains of *Escherichia coli*, *Klebsiella* and *Salmonella* [8,9] and drop after antibiotic treatment that removes Gram-negative enteric flora [10]. Interestingly, the binding of anti- αGal antibodies to blood Gram-negative bacteria isolates was higher than their binding to normal stool isolates [9]. In blood isolates, anti- αGal IgG antibodies may bind the capsular polysaccharide, increasing the alternative complement pathway lysis of the microorganism, or the lipopolysaccharide that makes the bacteria resistant to lysis [9].

We have previously described higher titers of natural anti- αGal IgM antibodies in some patients at the time of initiation of renal replacement therapy, which correlated with increased serum levels of TNF-α [11]. This condition predicted later risk for enteric peritonitis in peritoneal dialysis patients and mortality in both peritoneal dialysis and hemodialysis patients [11]. In addition, we have recently shown that boosted TI xenoantibodies in rats by exposure to hamster or pig antigens cross-react to *Enterococcus faecalis* by targeting melibiose and L-rhamnose carbohydrates [12]. This circumstance was associated with reduced survival of these animals after CLP, which was not observed with the generation of IgG antibodies. To elucidate the mechanisms underlying these findings, we analyzed the xenoantibody and inflammatory responses caused by exposure to xenogeneic antigens and their impact on CLP-induced sepsis in a rat model. The production of xenoantibodies in rats is characterized by the predominant initial expansion of TI natural IgM antibodies (first week), followed by the generation of T-cell-dependent adaptive IgG antibodies (days 21 to 28) [6]. This allows the assessment of the impact of the TI xenoantibodies in the outcome of sepsis.

## Material and Methods

### Animals

Lewis rats (200–250 g weight) and Golden Syrian hamsters (100–150 g weight) were purchased from Interfauna Harlan Iberica SL (Barcelona, Spain). Animals were maintained at University of Barcelona (Bellvitge Campus) animal facility under controlled conditions of temperature (21 ± 1°C) and humidity (55 ± 5%), cycles of light/dark of 12/12 h, and with food and water given ad libitum. Mice were acclimatized for 1 week before entering the study. Animals were anesthetized by isoflurane inhalation for blood draw and injections: deep anesthesia (at 4%) for hamsters (cardiac puncture), middle (at 2%) for rat blood draw and light (at 1%) for rat blood injection. All animal procedures were supervised and approved by Bellvitge Biomedical Research Institute (IDIBELL) ethics committee for animal experimentation and the Catalonia Government (Permit Number: DMA 3225). The care and handling of the animals conforms to the *Guide for the Care and Use of Laboratory Animals* published by the US National Institutes of Health (NIH Publication n° 85–23 revised 1996) and the *European Agreement of Vertebrate Animal Protection for Experimental Use* (86/609).

### Rat immunization

To produce a pattern of predominantly TI anti-hamster xenoantibodies [6], three intraperitoneal (ip) injections of 1 ml of hamster blood were given on alternating days (days -5, -3 and -1 relative to CLP). Control animals were subjected to three ip injections of phosphate-buffered saline (PBS) on the same days. Hamster blood was collected heparinized from cardiac puncture, pooled and immediately injected ip into rats.

### Determination of xenoantibodies

IgM and IgG anti-hamster xenoantibodies were determined by flow cytometry, measuring only the surface-bound immunoglobulins. Lymphocytes obtained from the hamster spleen (1x10^6^ cells per sample) were incubated with test sera diluted 1/50 in PBS/1% bovine serum albumin (BSA) at 4°C for 30 minutes in a final volume of 100 μl in V-bottom 96-well microtiter plates (NUNC Denmark). After the first incubation, a wash with PBS/1% BSA was performed followed by a second incubation (4°C 30 minutes in the dark) with 100 μl of a mixture of both polyclonal secondary antibodies (goat F(ab')2 fragment anti-rat IgG (H+L) conjugated with dichloro triazinyl aminofluorescein (DTAF) [dilution 1/100] and goat F(ab')2 fragment anti-rat IgM (μ) conjugated with phycoerythrin (PE) [dilution 1/200] (Beckman Coulter Immunotech) in PBS/1% BSA. Finally, cells were washed, resuspended in PBS and transferred to FACS tubes. For the determination of fluorescence intensity, a FACSCalibur cytometer (BD Biosciences) was used with three detectors/photomultipliers, which detect the light emitted at 530 nm (FL1), 585 nm (FL2) and > 670 nm (FL3), along with the help of programs for acquisition and analysis (Cell Quest) and for verification (FACS Comp).

### CLP

CLP was performed as described elsewhere [13]. Briefly, Lewis rats were anesthetized with 3–4% isoflurane and under sterile conditions a 1–2 cm midline incision was made and the cecum was exteriorized and ligated (4–0 Safil Violet, B/Braum, Germany) distal to the ileocecal valve. To generate a low-grade sepsis, defined as ~ 0–15% mortality during the acute phase of sepsis, 25% of the cecum (~ 10 mm) was ligated and punctured twice with a 19-gauge needle. The abdomen was closed in two layers and animal recovery was facilitated by keeping the animal on a thermal blanket. Access to water and food was facilitated 2 h after surgery when animals were placed in their corresponding cages. Body weight, mobility, food intake, cutaneous features and respiratory frequency of animals were monitored twice a day over a period of 15 days, providing a score (S1 Table) that led to different corrective measures depending on animal status (S2 Table).

### Blood bacterial load

Blood collected after cardiac puncture was serially diluted and immediately plated on Trypticase Soy Agar II plates supplemented with 5% Sheep Blood (Becton Dickinson). Colony-forming units (CFU) were counted after 24 h incubation at 37°C.

### Blood hematology and serum biochemistry

One ml of blood was collected from the tail vein at different times, centrifuged (2 x 10 min, 5000 rpm) and the sera were frozen at -20°C. ALT, AST, urea and triglycerides were measured according to protocols of the International Federation of Clinical Chemistry by spectrophotometric analysis at the Veterinarian School of Barcelona Autonomous University. EDTA-anticoagulated blood (0.5 ml) was also obtained for hematological analysis by cytometry utilizing peroxidase staining (ADVIA 120 Hematology System, Siemens Healthcare), according to protocols of Veterinarian School of Barcelona Autonomous University.

### Cytokine/Chemokine Analysis

Rat serum was assessed for the presence of 33 cytokines including proinflammatory and anti-inflammatory cytokines, chemokines, growth factors and soluble cell receptors (S3 Table). Antibody array membranes (rat cytokine antibody array I; RayBiotech, Norcross, GA) were first blocked for 30 min with the provided blocking buffer, to which rat sera were subsequently added for a final 10-fold dilution of the sera. Membranes were then incubated for 2 h at room temperature with shaking. After addition of a biotin-conjugated anti-cytokine antibody mixture, the membrane was incubated with horseradish peroxidase-conjugated streptavidin and developed. Signal intensity was quantified with the Quantity One software (Bio-Rad Laboratories). The advantages of using this multiple cytokine analysis have been previously described [14]: 1) is a panel commercially available permitting easily replication studies; 2) allows the simultaneous analysis of cytokines, chemokines, soluble cell receptors and growth factors; 3) many of them have been engaged in the pathogenesis of sepsis; 4) includes cytokines with both pro- and anti-inflammatory activities allowing a large evaluation of immune responses.

### Power of the study and statistical analysis

We calculated that a sample size of 20 animals per group would provide the appropriate power (1-β = 0.8) to identify a significant (α = 0.05) reduction in rat survival after CLP from 90% to 50%. The results are expressed as the mean ± standard error of the mean (SEM). The differences for each group at different times were compared by one-way ANOVA for repeated measures with the Bonferroni correction. Survival data were compared by the log-rank test. To evaluate the interactive and independent effects on cytokine expression in three groups (control-TI, TI-alive, TI-death) and at three times (days -5, 0, 2), a repeated-measures two-way ANOVA was used. As the interaction term was significant (p<0.001), individual One-way ANOVAs for repeated measures with the Bonferroni correction were performed for each cytokine to assess differences at the three times evaluated. Statistical results were considered significant if p-value<0.05. Statistical analyses were performed using R 2.15: A language and environment for statistical computing (R Foundation for Statistical Computing, Vienna, Austria).

## Results

### Impact of anti-hamster xenoantibodies on Lewis rat survival after CLP

**Fig 1:**
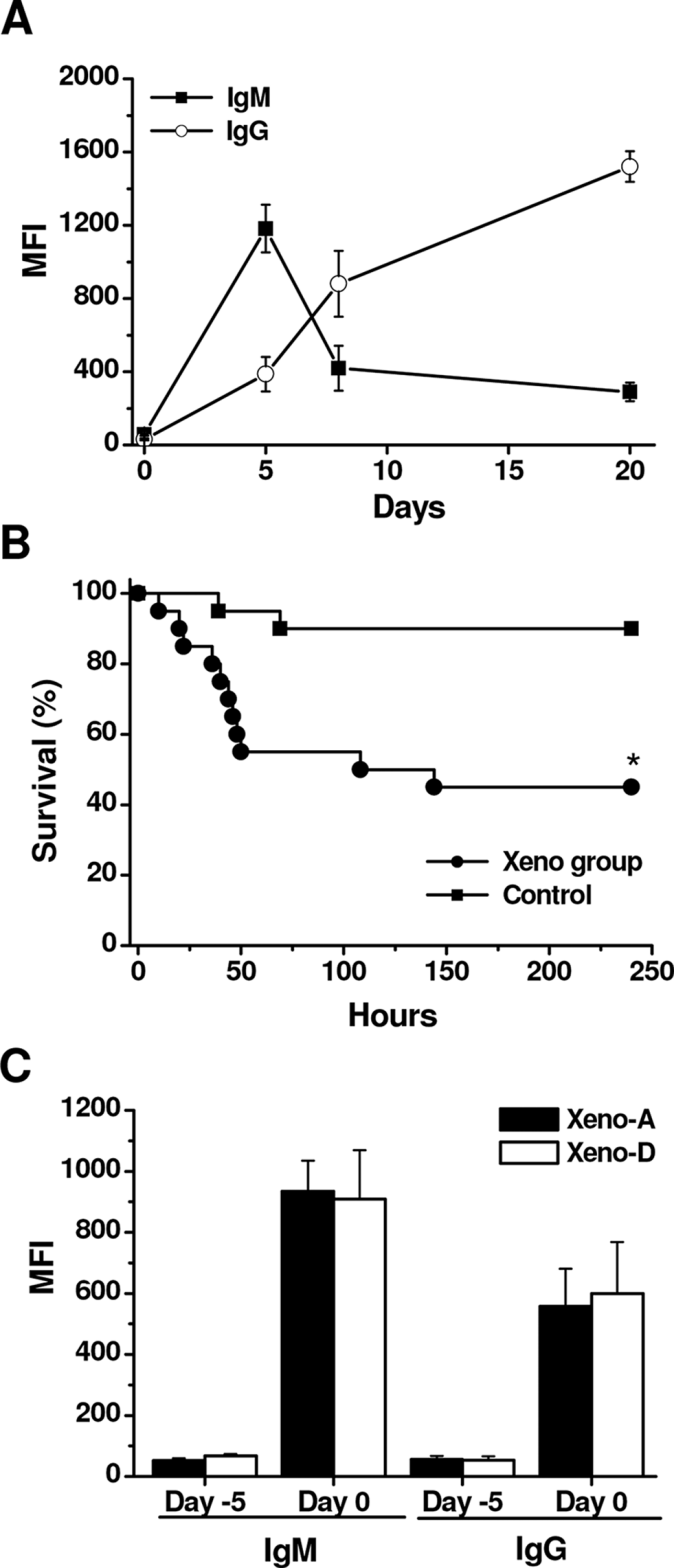
Generation of rat anti-hamster xenoantibodies and their impact on sepsis survival after CLP. (***A***) Levels of anti-hamster IgM and IgG xenoantibodies in Lewis after three injections of hamster blood, with one injection administered every other day. Mean ± SEM of 5 animals per group. MFI: Mean fluorescence intensity. (***B***) Percent survival of Lewis rats with and without enhanced anti-hamster xenoantibodies submitted to CLP (n = 20 per group; *p<0.01). (***C***) Level of anti-hamster IgM and IgG xenoantibodies at baseline (day—5) and before CLP (day 0) in Lewis rats immunized with hamster blood and subsequently submitted to CLP that survived (Xeno-A; n = 9) or died (Xeno-D; n = 11) after the procedure. Values are the mean ± SEM.

Exposure of Lewis rats to three injections of hamster blood, with one injection administered every other day, caused an increase of IgM and IgG anti-hamster xenoantibodies at day 5 compared to control animals. On this day, the average level of IgM was three-fold higher than IgG xenoantibodies (Fig 1A), following contrary patterns thereafter to day 20 when the average level of IgG was 5-fold higher than IgM (Fig 1A). No change was observed in the level of IgM and IgG xenoantibodies with PBS injections in control animals (data not shown). CLP sepsis was performed in Lewis rats on day 5 after xenoimmunization or the corresponding control injections. Augmentation of anti-hamster xenoantibodies was directly associated with lower survival after CLP compared to controls (45% vs. 90%, p<0.01) (Fig 1B). No differences were observed in the levels of anti-hamster IgM and IgG xenoantibodies before CLP between rats that died after the procedure and those that did not (Fig 1C). Bacterial loads were also assessed 24 h after CLP. However, no bacteria were detected in blood samples after CLP either after boosting anti-hamster xenoantibodies or not, despite the presence of signs of inflammation and abscesses in the abdomen.

### Changes in body weight after xenoimmunization and CLP

**Fig 2:**
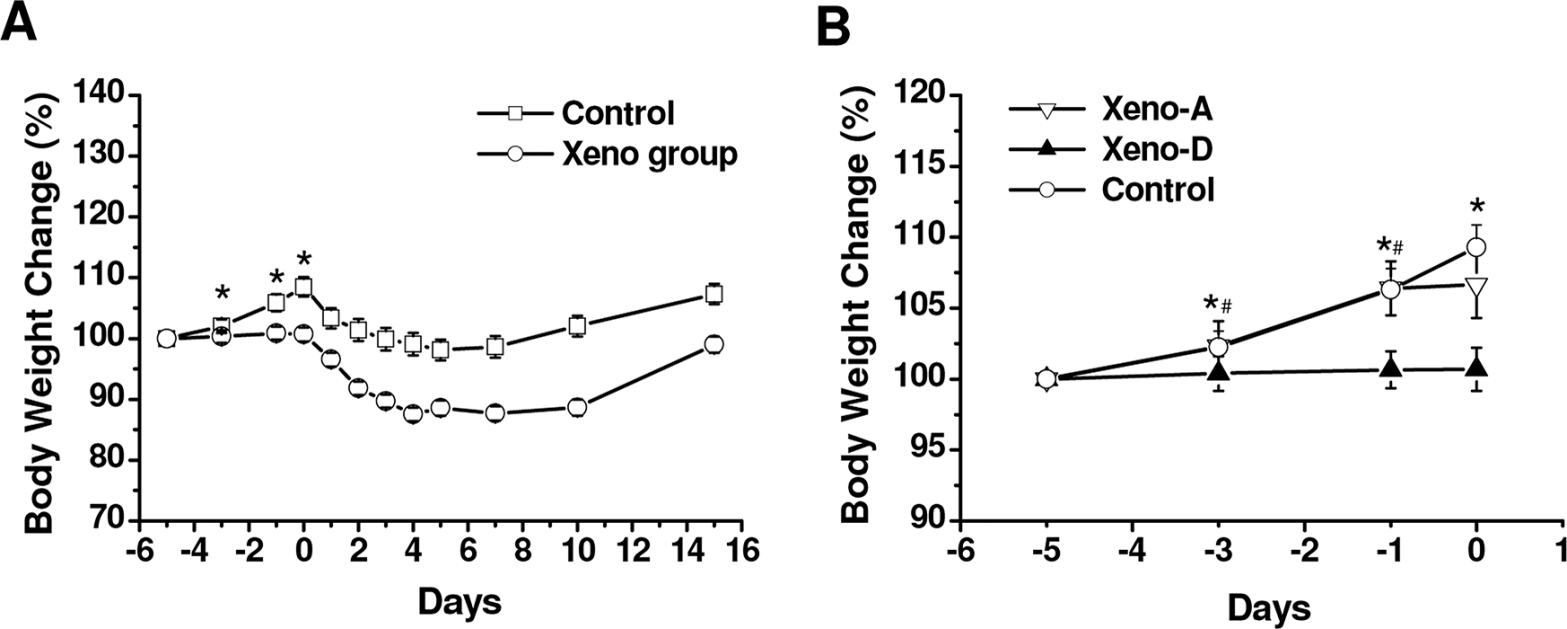
Change in body weight. (***A***) Body weight before and after CLP (day 0) with or without enhancement of anti-hamster xenoantibodies (n = 20 per group from day -5 to 0, n = 18 in control from day 3 to 10, and n = 9 in Xeno group from day 6 to 10). (***B***) Body weight before CLP in animals with boosted anti-hamster xenoantibodies that survived (Xeno-A; n = 9) or died (Xeno-D; n = 11) after CLP. (*p<0.05 compared to control; #p<0.05 compared to Xeno-A).

We evaluated the effects of xenoimmunization and CLP on the body weight of Lewis rats. The weight of control rats increased from day -5 to 0 (day of CLP), whilst there was no change in xenoimmunized animals (Fig 2A). The differences in weight gain between control and rats with boosted xenoantibodies were significant on days -3, -1 and 0 (time of CLP) (Fig 2A). After CLP, there was a decrease over a period of 5 days in the body weight of both control and xenoimmunized animals that survived the procedure, with an increase in body weight thereafter (Fig 2A). When analyzed separately, animals with raised anti-hamster xenoantibodies that died after CLP for low-grade sepsis did not change in body weight from day -5 to 0 (Fig 2B). In contrast, rats with increased xenoantibodies that later survived CLP for low-grade sepsis had a similar weight gain to that of controls from day -5 to 0 (Fig 2B).

### White blood cell (WBC) and biochemical changes with xenoimmunization and CLP

**Fig 3:**
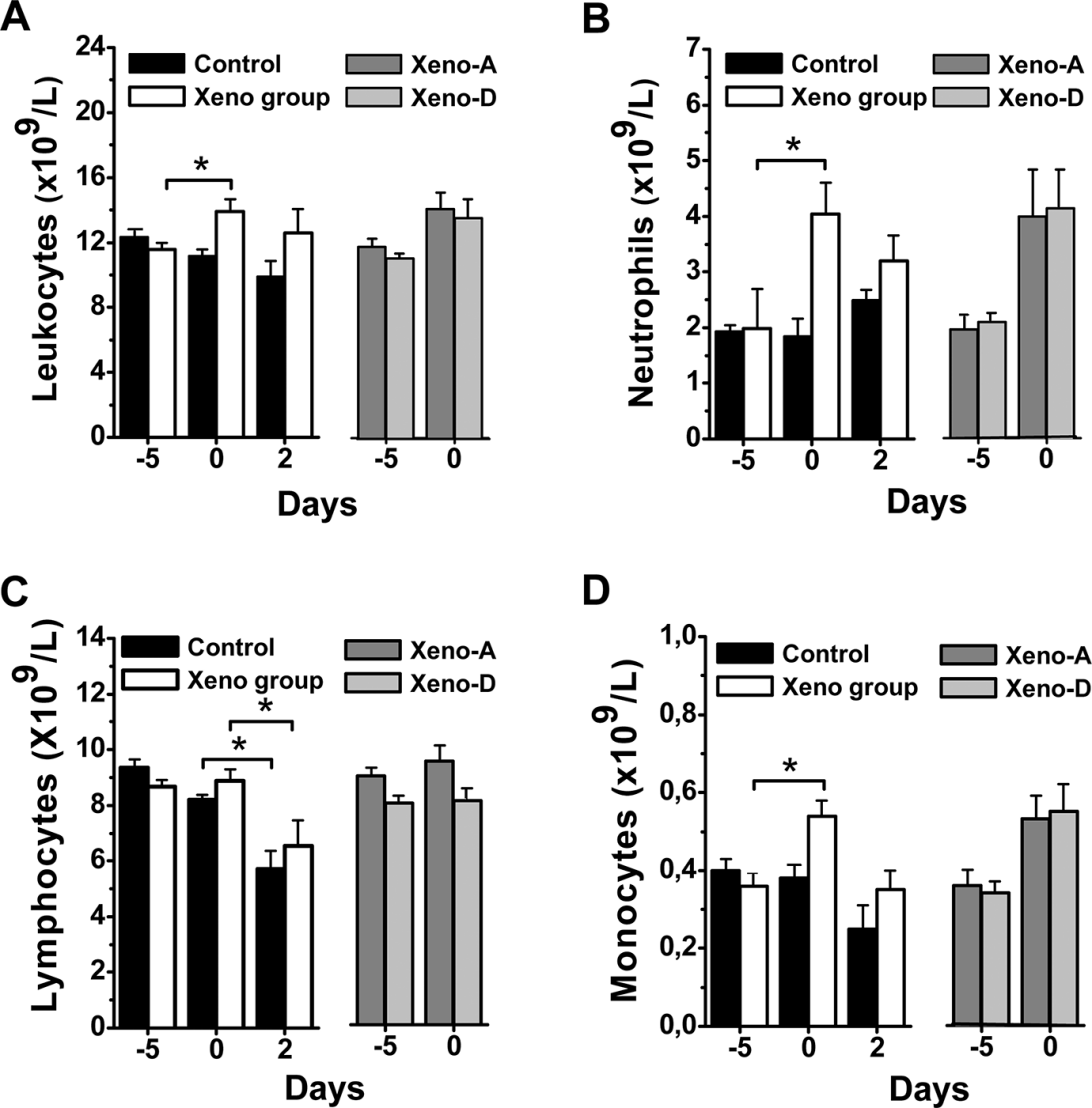
Absolute leukocyte counts present in peripheral blood. Total leukocytes (***A***), neutrophils (***B***), lymphocytes (***C***) and monocytes (***D***), on days -5, 0 and 2 of CLP in animals with and without enhancement of anti-hamster xenoantibodies (n = 20 per group) and on days -5 and 0 in rats with boosted anti-hamster xenoantibodies that survived (Xeno-A; n = 9) or died (Xeno-D; n = 11) after CLP. (*p<0.05).

Enhancement of anti-hamster xenoantibodies was associated with a significant augmentation in total blood leukocytes, neutrophils and monocytes just prior to CLP (day 0) compared to baseline (day -5), whilst no change was observed in lymphocyte count (Fig 3A–3D). CLP caused a significant reduction of lymphocytes in rats with augmented xenoantibodies and in control rats (Fig 3C).

**Fig 4:**
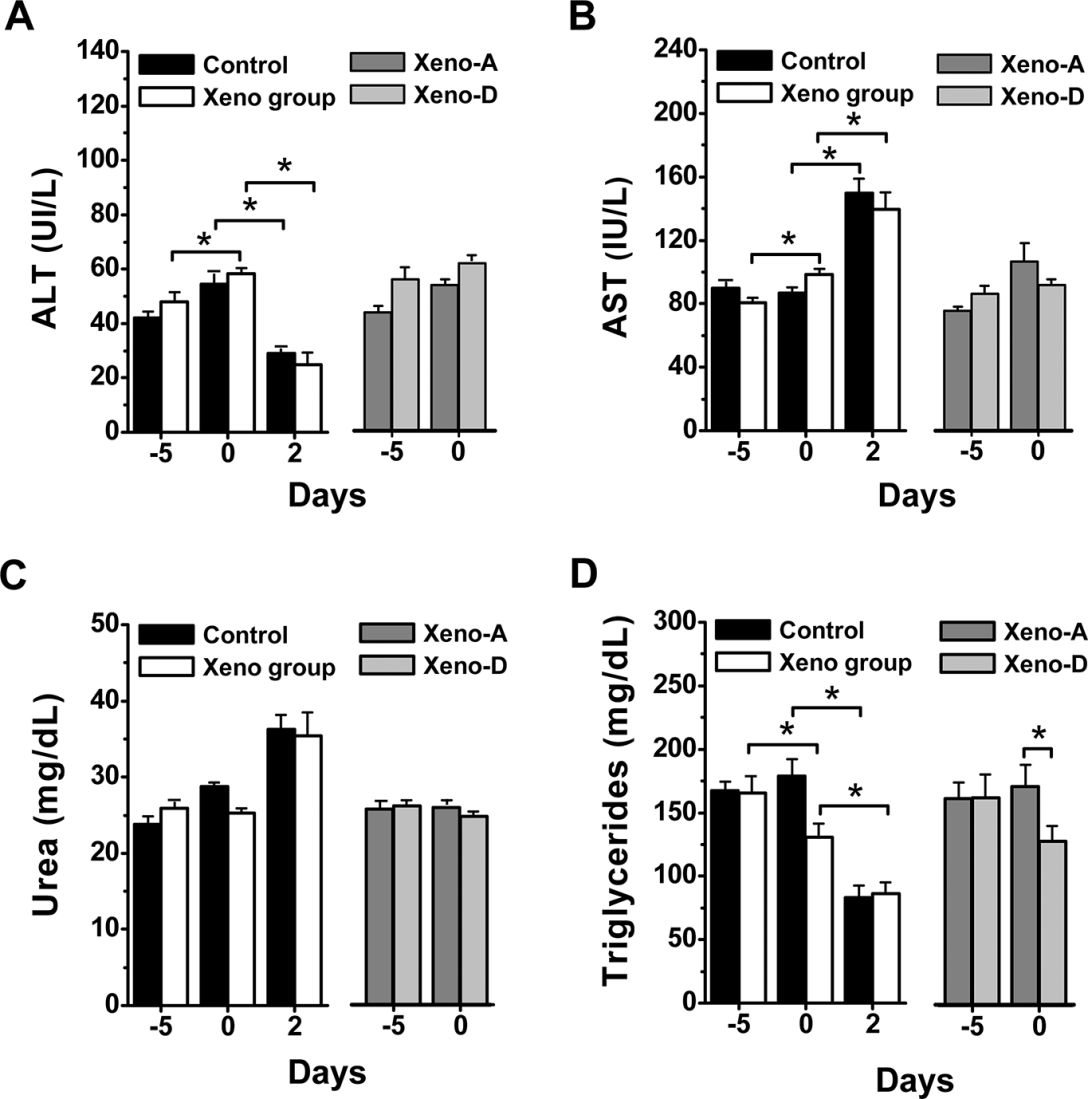
Clinical chemistry determinations in peripheral blood. ALT (***A***), AST (***B***), urea (***C***) and triglycerides (***D***), on days -5, 0 and 2 of CLP in animals with and without enhancement of anti-hamster xenoantibodies (n = 20 per group) and on days -5 and 0 in rats with boosted anti-hamster xenoantibodies that survived (Xeno-A; n = 9) or died (Xeno-D; n = 11) after CLP. (*p<0.05).

The increase of anti-hamster xenoantibodies in rats also generated changes in biochemical parameters, which included an augment of alanine and aspartate aminotransferases (ALT and AST), along with a decrease in triglycerides prior to CLP compared to baseline (Fig 4A–4D). CLP resulted two days later in a reduction in ALT and triglycerides, along with an increase of AST both in rats with boosted xenoantibodies and controls (Fig 4A–4D). Animals with raised xenoantibodies that survived CLP showed higher levels of triglycerides at the time of the procedure compared to animals that died (Fig 4D).

### Effect of xenoimmunization and CLP on blood cytokines

**Fig 5:**
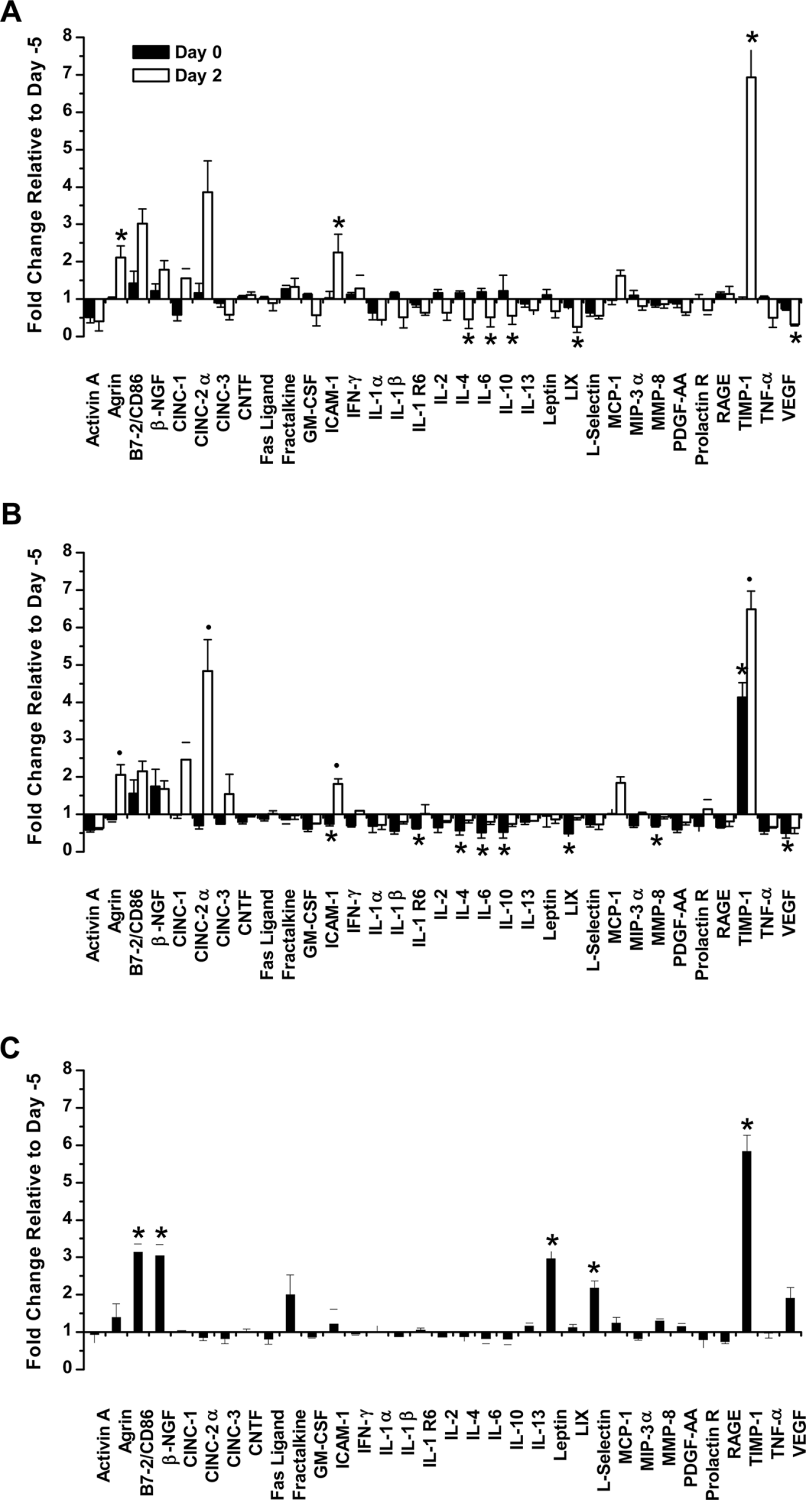
Cytokine concentrations. Fold changes of 33 cytokines on days -5 (baseline), 0 (before CLP; black column) and 2 (two days after CLP; white column). (***A***) Control; (***B***) animals with increased anti-hamster xenoantibodies that survived CLP (Xeno-A); (***C***) animals with boosted anti-hamster xenoantibodies that died after CLP (Xeno-D). Mean ± SEM of 3 animals per group. (*p<0.05 compared to day -5;#p<0.05 compared to days -5 and 0).

To investigate further the effects of xenoimmunization and CLP, we analyzed the blood pattern of 33 cytokines (mediators of cell signaling) in rats with increased anti-hamster xenoantibodies that survived or died after CLP, and in control animals. No change was observed for any cytokine in the control group at the time of CLP (day 0) compared to baseline (day -5) (Fig 5A). Xenoimmunization of animals that subsequently survived CLP produced at pre-CLP time significant decreases compared to baseline in ICAM-1, IL-1 R6, IL-4, IL-6, IL-10, LIX, MMP-8, and VEGF along with a significant augment of TIMP-1 (Fig 5B). In contrast, the boosting of xenoantibodies in animals that died after CLP did not result in any reduction of cytokines at the time of the procedure compared to day -5 and was associated with significant increases of B7-2/CD86, β-NGF, leptin, L-selectin and TIMP-1 (Fig 5C).

Two days after CLP, control rats showed a significant decrease in IL-4, IL-6, IL-10, LIX, and VEGF, along with increases in agrin, ICAM-1 and TIMP-1, compared to day 0 (pre-CLP) (Fig 5A). In animals with boosted xenoantibodies that survived CLP, there was a significant increase at 48 h post-CLP in agrin, CINC-2α, ICAM-1 and TIMP-1 compared to baseline and pre-CLP samples, but no major changes for the other cytokines (Fig 5B).

## Discussion

The present study confirmed that exposuring Lewis rats to hamster cells resulted in mainly expanded TI natural xenoantibodies, systemic alterations and impaired survival after CLP-induced sepsis. The exacerbated mortality was not directly influenced by the amount of xenoantibodies. Similar levels of IgM and IgG anti-hamster xenoantibodies at the pre-CLP time point were observed both in xenoimmunized rats that survived and those dying after the procedure. Likewise, the data did not show an association between increased mortality and a particular effect of xenoantibodies on blood bacterial isolates. No blood bacterial loads were observed after this CLP model regardless of whether xenoantibodies were boosted or not. The lack of blood bacteria isolates after CLP in our model indicates that the enhancement of anti-melibiose and L-rhamnose antibodies reactive to *E*. *faecalis* caused by the hamster blood exposure is not responsible for the observed increased mortality [11]. However, it does not rule out an impairment of sepsis if bacteremia is present and caused by microorganisms expressing carbohydrate antigens targeted by natural antibodies, as has been suggested for αGal and poly-N-acetyl glucosamine antigens [9,15].

Xenoimmunization to boost mainly TI anti-hamster antibodies in rats produced a systemic inflammatory response at the pre-CLP time point, as evidenced by leukocytosis resulting from an increase in blood neutrophils and monocytes, along with increases in ALT and AST. There was also a lack of weight gain and a drop in triglyceride levels, though no animal died before CLP. Interestingly, the impairment of the nutritional status coinciding with the non-infectious systemic inflammation affected mostly those rats that subsequently died after CLP. A process of peritonitis can be suspected in these animals. The malnutrition signs were even more pronounced after CLP in these animals, affecting also xenoimmunized animals that survived CLP and controls. CLP did not change blood leukocyte counts compared to pre-CLP besides the lymphopenia 48 h after the procedure, as was previously observed [16], both in xenoimmunized rats and controls. However, there was an increase in serum AST and a decrease in ALT. To our knowledge, this is the first time that a decrease in serum ALT levels has been reported after CLP, which in our study involved xenoimmunized animals and controls. Previous studies describe a significant increase in ALT resulting from liver damage after CLP, particularly in moderate-severe sepsis [17], which returns to baseline levels by day 4 in low-severity models. The decrease in ALT after CLP may reflect the impairment of the nutritional status of the animals, as has been suggested in humans [18], though it was not observed initially in xenoimmunized rats with lack of weight gain.

Our investigations did not include the phenotype assessment of the diverse leukocytes involved in the inflammatory response of xenoimmunization and CLP, which certainly is a shortcoming of the study. However, immune cells implement their tasks primarily through the secretion of cytokines that mediate functions such as direct killing, self-renewal, recruitment of other cells, and promotion or inhibition of inflammation. These cytokines may be produced differently by a particular cell and not always correlates with a specific cell phenotype [19]. Therefore, we consider that a high-throughput multiplex cytokine assay could also characterize the magnitude and quality of the immune response. In this study, CLP in rats without previous xenoimmunization led to minimal mortality and (on day two) the augmentation in blood of agrin (soluble cell receptor), ICAM-1 (soluble cell receptor) and TIMP-1 (metalloproteinase with pleiotropic activities). However, there was a predominant pattern of reduction of cytokines that included IL-4, IL-6, IL-10, LIX and VEGF (pro-inflammatory and anti-inflammatory cytokines, chemokines and growth factors). The impaired production of cytokines has been previously reported with non-lethal CLP [20], which contrasts to the elevated cytokine levels observed in sepsis of increased mortality [16,21,22]. In these studies that assessed only particular cytokines, mortality after CLP related to the augmentation of only proinflammatory cytokines such as IL-6 [16,23,24], or both pro- and anti-inflammatory cytokines [22,25]. However, when a cytokine profile using a multiplex cytokine measurement is employed as in our study, patterns including the simultaneous augment and reduction of different cytokines are detected [14,26,27], similar to what we observed in our analysis.

Xenoimmunized rats that later survived CLP showed a more clear profile of blood cytokine reduction at the pre-CLP time point, which included cytokines which also decreased after CLP in control animals along with others such as ICAM-1, IL-1R6 and MMP-8, being TIMP-1 the only cytokine that was augmented. In these rats all the cytokine levels increased in response to CLP, displaying a pattern with no cytokine decreases. A similar cytokine profile and outcome with secondary bacterial infections has been described after peritoneal lypopolysaccharide (LPS) challenge in animals previously exposed to peritoneal bacterial products or cytokines such as tumor necrosis factor (TNF) [20]. Unfortunately, no information was provided about the impact of the latter procedures in the cytokine profile before LPS challenge. Suppression of TLR and inflammatory responses has been described with IgM natural antibodies and in endotoxin tolerance [28–30]. This condition is a phenomenon observed after exposure to low concentrations of LPS that produce a transient unresponsive state to further challenges with endotoxin [31,32]. Thus, xenoantibodies and/or immune complexes generated by xenoimmunization may provide a predominant profile of negative regulation of TLR-triggered inflammatory responses, leading to a response similar to that of non-lethal CLP.

In contrast, no cytokine was reduced at the pre-CLP time point in xenoimmunized animals that died after CLP. Instead, there were increases in B7-2/CD86 (soluble cell receptor), β-NGF (growth factor, adipokine), leptin (adipokine), L-selectin (soluble cell receptor) and TIMP-1. Interestingly, the increase of cytokines in these rats included leptin and β-NGF that are identified as adipokines since they are secreted by adipose tissue [33]. Leptin has been shown to be elevated in early stages of inflammation and in the exacerbation of sepsis mortality [34,35]. There is also evidence that leptin is produced by intraperitoneal adipocytes [36,37], and that increased concentrations of this and other adipokines have a very high sensitivity and specificity for an early diagnosis of peritonitis in patients undergoing peritoneal dialysis [38]. In our study, they may have contributed to the lack of weight gain and metabolic disorders observed in animals during xenoimmunization [39], and later mortality after CLP.

This study has failed to show a particular effect of boosted xenoantibodies in the increased mortality of CLP. However, it indicates that the inflammatory profile before infectious injuries is crucial to the response that will occur afterwards. This concept was already recognized in severe non-infectious situations, such as trauma or hemorrhage, along with the impact of these conditions on the development of sepsis [40,41]. Likewise, it may be extended to non-critical conditions or even, apparently, to normal circumstances. Using single cytokine analysis, higher pre-infection levels have been associated with an elevated risk of community-acquired pneumonia requiring hospitalization and mortality in dialysis patients [11,42]. In the latter case, it was also associated with an increase of natural IgM xenoantibodies. Also, the metabolic syndrome is associated with increased levels of adipokines [43], which in our study showed a particular involvement in increasing the severity of sepsis. Overall, our results suggest that preventive strategies acting on the inflammatory status of susceptible patients prior to infection may help averting the harmful consequences of sepsis

## Conclusions

Enhancement of predominantly natural anti-hamster xenoantibodies in Lewis rats with a xenoimmunization protocol was associated with increased mortality from low-grade sepsis after CLP. The impairment of sepsis survival in rats could not be correlated with the level of xenoantibodies or bacteremia. However, it was associated with a lack of weight gain during xenoimmunization and a cytokine profile at the time of CLP characterized by increased levels of cytokines, particularly adipokines such as leptin and β-NGF. This profile contrasted with the reduction in cytokine levels observed in xenoimmunized rats that gained weight and survived CLP. Thus, the outcome of sepsis appears to depend more on the cytokine profile existing before sepsis than that resulting from the condition, with a special harmful effect of adipokines.

## Supporting Information

S1 Table Scoring to evaluate rat body weight, general aspect, self-mutilation or signs of pain and response to stimulus. (DOC)

S2 Table Corrective measures applied to rats depending on final score. (DOC)

S3 Table Cytokine class and effect included in array analysis (from http://copewithcytokines.de). (DOC)
