# Supplementary material for: Divergence of the Response Induced by Xenogenic Immunization in the Sepsis Survival of Rats
Source: PLoS One. 2015 May 18;10(5):e0125472. doi: 10.1371/journal.pone.0125472 (PMC4436005; doi:10.1371/journal.pone.0125472)
Supplement: S1 Table — (DOC) [file pone.0125472.s001.doc]

**Table S1. Scoring to evaluate rat body weight, general aspect, self-mutilation or signs of pain and response to stimulus.**

| **Parameter** | **Score** | |
| --- | --- | --- |
| *Body weight loss* | none | 0 |
|  | < 10% | 1 |
|  | Between 10% and 20% | 2 |
|  | Between 10% and 20% and modification of the feces | 3 |
|  | > 20% | 4 |
|  | No food and water intake  (Immediately euthanized) | 5 |
| *General appearance* | Normal | 0 |
|  | Dull and/or bristling coat | 1 |
|  | Dull and/or bristling coat and/or ocular or nasal secretions | 2 |
|  | Abnormal posture | 3 |
| *Self-mutilation or signs of pain* | Yes | 3 |
|  | No | 0 |
| *Response to stimulus* | Normal | 0 |
|  | Minor changes | 1 |
|  | Reduced mobility and/or inactive | 2 |
|  | Aggressive or comatose, convulsions and/or temblors | 3 |
