# Supplementary material for: Divergence of the Response Induced by Xenogenic Immunization in the Sepsis Survival of Rats
Source: PLoS One. 2015 May 18;10(5):e0125472. doi: 10.1371/journal.pone.0125472 (PMC4436005; doi:10.1371/journal.pone.0125472)
Supplement: S2 Table — (DOC) [file pone.0125472.s002.doc]

**Table S2.** Corrective measures applied to rats depending on final score.

| **Score** | **Corrective measure** |
| --- | --- |
| 0 to 5 | Direct supervision (twice a day) |
| 5 to 9 | Direct supervision (twice a day) and buprenorphine administration (0.05 mg/kg/8 h) |
| 9 to 12 | Direct supervision (twice a day), buprenorphine administration (0.1 mg/kg/8 h) and euthanasia^a^ in the following 24 h |
| ≥12 | Immediate euthanasia^a^ |

^a^ Euthanasia procedure was established following the European Directive on the protection of animals used for scientific purposes (2010/63/EU). Briefly, the animals were placed in a gauzy euthanasia chamber where it can be easily visible. The chamber was not overcrowded by combining multiple cages, allowing all animals to make normal postural adjustments. The animals were euthanized by inhalation in an atmosphere of 100% carbon dioxide (CO_2_, flow rate of 50 L/min during 10 minutes) with early loss of consciousness and minimum pain, suffering and distress. Death was never considered as a human endpoint.
