# Supplementary material for: Divergence of the Response Induced by Xenogenic Immunization in the Sepsis Survival of Rats
Source: PLoS One. 2015 May 18;10(5):e0125472. doi: 10.1371/journal.pone.0125472 (PMC4436005; doi:10.1371/journal.pone.0125472)
Supplement: S3 Table — (DOC) [file pone.0125472.s003.doc]

**Table S3.** Cytokine class and effect included in array analysis (from [http://copewithcytokines.de](http://copewithcytokines.de/))

| **Cytokine** | **Class** | **Effect** |
| --- | --- | --- |
| Activin A | Growth factor | Inhibitory |
| Agrin | Soluble cell receptor | Unclear |
| B7-2/CD86 | Soluble cell receptor | Unclear |
| β-Nerve growth factor (NGF) | Growth factor, adipokine | Pro- and Anti-inflammatory |
| Cytokine-induced neutrophil chemoattractant (CINC)-1 | Chemokine | Pro-inflammatory |
| CINC-2 α | Chemokine | Pro-inflammatory |
| CINC-3 | Chemokine | Pro-inflammatory |
| Ciliary neurotrophic factor (CNTF) | Growth factor | Differentation effects |
| Fas-ligand | Soluble cell receptor | Anti-inflammatory |
| Fractalkine | Chemokine | Pro-inflammatory |
| Granulocyte-macrophage colony stimulating factor (GM-CSF) | Growth factor | Pro-inflammatory |
| Intercellular adhesion molecule (ICAM)-1 | Soluble cell receptor | Anti-inflammatory |
| Interferon (IFN)-γ | Cytokine | Pro-inflammatory |
| Interleukin (IL)-1 α | Cytokine | Pro-inflammatory |
| IL-1 β | Cytokine | Pro-inflammatory |
| IL-1 receptor (R) 6 | Soluble cell receptor | Anti-inflammatory |
| IL-2 | Cytokine | Pleiotropic |
| IL-4 | Cytokine | Anti-inflammatory |
| IL-6 | Cytokine | Pro-inflammatory |
| IL-10 | Cytokine | Anti-inflammatory |
| IL-13 | Cytokine | Anti-inflammatory |
| Leptin | Adipokine | Pro-inflammatory |
| LPS induced CXC chemokine (LIX) | Chemokine | Pro-inflammatory |
| L-Selectin | Soluble cell receptor | Anti-inflammatory |
| Monocyte chemoattractatn protein (MCP)-1 | Chemokine | Pro-inflammatory |
| Macrophage inflammatory protein (MIP)-3α | Chemokine | Pro-inflammatory |
| Matrix Metalloproteinase (MMP)-8 | Chemokine | Pro-inflammatory |
| Platelet derived growth factor (PDGF)-AA | Growth factor | Pro-inflammatory |
| Prolactin-receptor (R) | Soluble cell receptor | Anti-inflammatory |
| Receptor for advanced glycosilation end products (RAGE) | Soluble cell receptor | Anti-inflammatory |
| Tissue inhibitor of metalloproteinases (TIMP)-1 | Growth factor | Pleiotropic |
| Tumor necrosis factor (TNF)-α | Cytokine | Pro-inflammatory |
| Vascular endothelial growth factor (VEGF) | Growth factor | Pro-inflammatory |
